# Supplementary material for: Engineering Nanofiber Scaffolds with Biomimetic Cues for Differentiation of Skin-Derived Neural Crest-like Stem Cells to Schwann Cells
Source: Int J Mol Sci. 2022 Sep 16;23(18):10834. doi: 10.3390/ijms231810834 (PMC9504850; doi:10.3390/ijms231810834)
Supplement: Supplementary file 1 [file ijms-23-10834-s001.zip › ijms-1790637-supplementary.pdf]

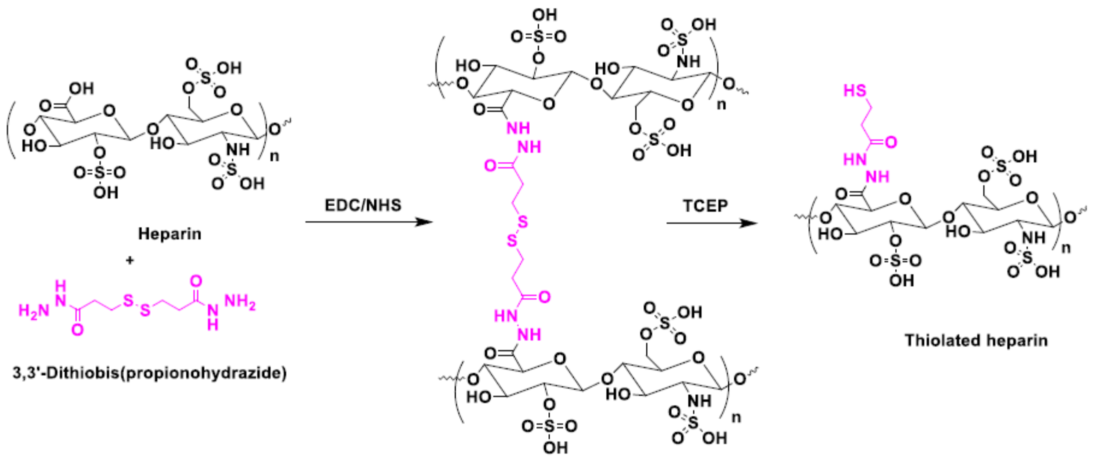

Figure S1. EDC-NHS mediated thiolation of heparin (Hep) via amidation reaction. To synthesize Hep-thiol, carboxyl groups of heparin are modified by dithiol (S-S) group containing hydrazide molecule via EDC-NHS chemistry which is later reduced by TCEP to introduce thiol group (-SH) in the structure.

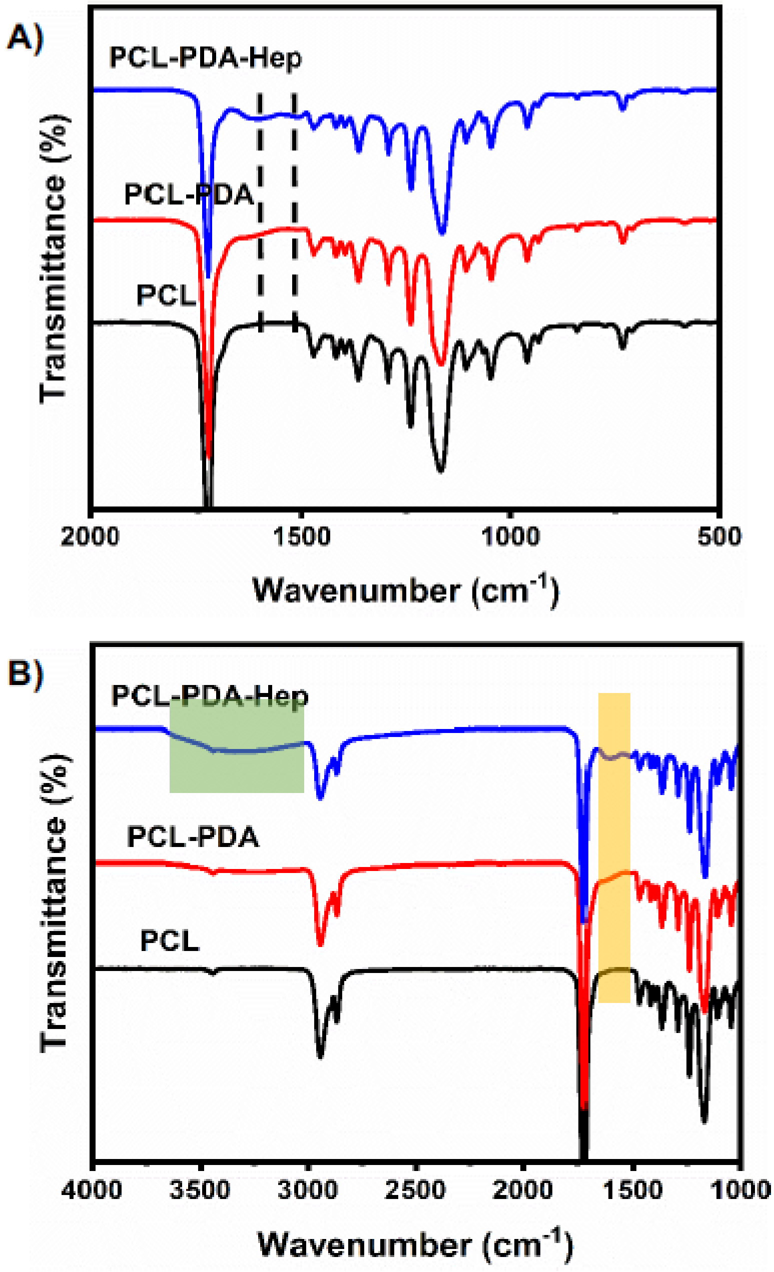

Figure S2. FTIR spectra of PCL, PCL-PDA, and PCL-PDA-Hep at wavenumber ranges: (A) 2000 – 500 cm^{-1} and (B) 4000 – 1000 cm^{-1}.

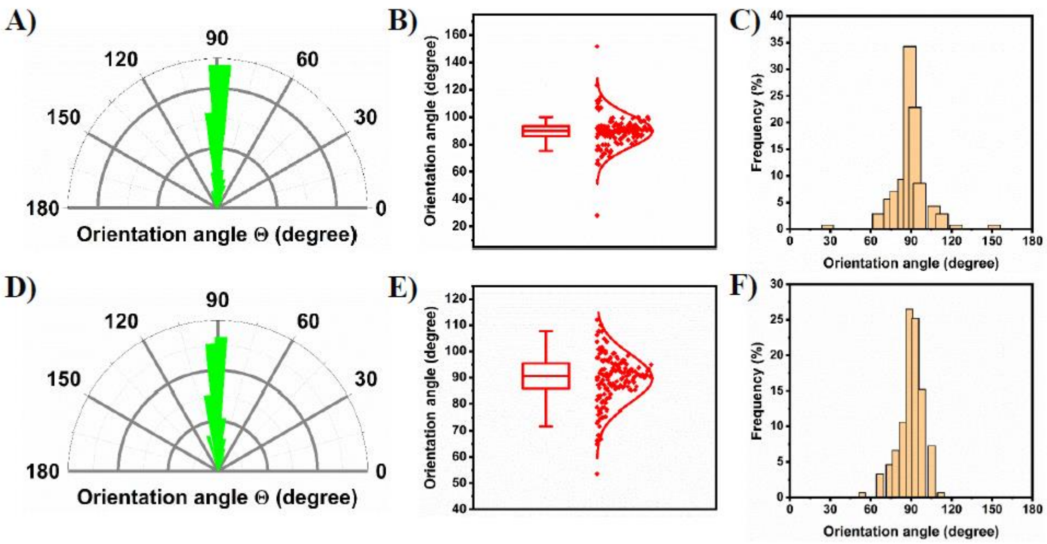

Figure S3. Nanofiber orientation. Orientation angles for PCL (A-C) and PCL-PDA-Hep (D-F) fibers were measured using image J software. **(**A, D) orientation angle measurements; (B, E) Orientation angle distribution and mean orientation angles; (C, F) percent frequency of fibers at different orientation angles; orientation angle bin: 5 degrees.

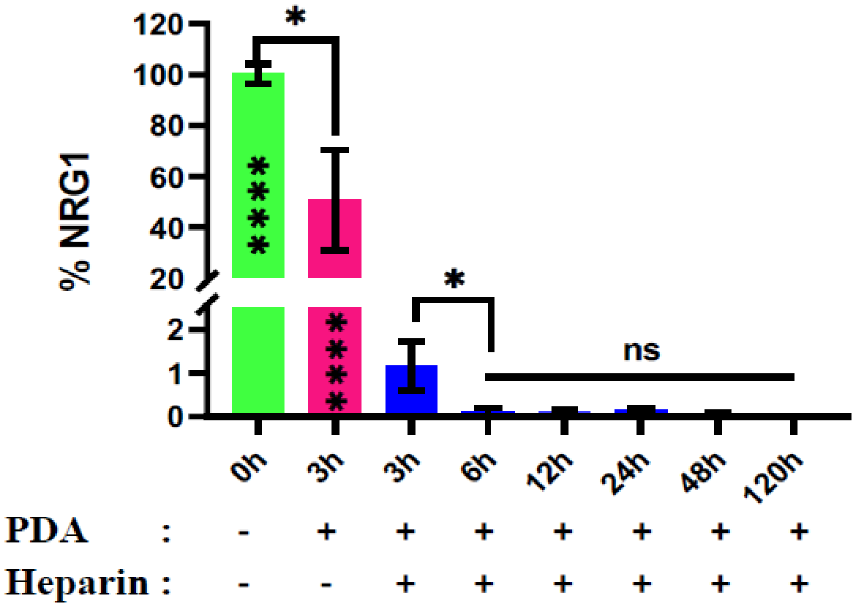

Figure S4. Release kinetics of the immobilized NRG1 from the PCL fiber surface.Release of NRG1 in the media at different time points after immobilization on the fibers. Data are presented as Mean ± SD, One-way analysis of variance (ANOVA) with Tukey post hoc test. n=3 independent experiments. nsP (≥0.05), *P (<0.05), ****P (<0.0001).
